# Early Sex Differences in the Immune-Inflammatory Responses to Neonatal Ischemic Stroke

**DOI:** 10.3390/ijms20153809

**Published:** 2019-08-04

**Authors:** Sonia Villapol, Valerie Faivre, Pooja Joshi, Raffaella Moretti, Valerie C. Besson, Christiane Charriaut-Marlangue

**Affiliations:** 1 Center for Neuroregeneration, Department of Neurosurgery, Houston Methodist Research Institute, Houston, TX 77030, USA; 2 U1141 NeuroDiderot, INSERM, Université Paris Diderot, Sorbonne Paris Cité, Hôpital Robert Debré, 48 Boulevard Sérurier, 75019 Paris, France; 3 EA4475–Pharmacologie de la Circulation Cérébrale, Faculté de Pharmacie de Paris, Université Paris Descartes, Sorbonne Paris Cité, 4 avenue de l’Observatoire, 75006 Paris, France; 4 UMR-S1144–Optimisation Thérapeutique en Neuropsychopharmacologie, Faculté de Pharmacie de Paris, Université Paris Descartes, Sorbonne Paris Cité, 4 avenue de l’Observatoire, 75006 Paris, France

**Keywords:** lesion, macrophages, microgliosis, neonatal stroke, neuroinflammation, neuronal loss, sex differences

## Abstract

We recently reported that neonatal ischemia induces microglia/macrophage activation three days post-ischemia. We also found that female mice sustained smaller infarcts than males three months post-ischemia. The objective of our current study was to examine whether differential acute neuroinflammatory response and infiltrated immune cells occurs between male and females after three days post-ischemia. Permanent middle cerebral artery occlusion was induced in male and female postnatal 9-day-old (P9) mice, and mice were sacrificed three days after ischemia. Brains were analyzed for mRNA transcription after microglia magnetic cell sorting to evaluate M1 and M2 markers. FACS analysis was performed to assess myeloid infiltration and microglial expression of CX3 chemokine receptor 1 (CX3CR1). Inflammatory cytokine expression and microglia/macrophage activation were analyzed via in situ hybridization combined with immunofluorescence techniques. Lesion volume and cell death were measured. An increase in microglia/macrophages occurred in male versus female mice. The cells exhibited amoeboid morphology, and *TNFα* and *ptgs2* (Cox-2) genes were more expressed in males. More myeloid cell infiltration was found in male versus female brains. However, we did not observe sex-dependent differences in the injured volume or cell death density. Our data show that sex differences in the acute microglial and immune responses to neonatal ischemia are likely both gene- and region-specific.

## 1. Introduction

In the perinatal period (between birth and 28 days of life), arterial ischemic stroke occurs in 1/2800 to 1/5000 live births and mainly affects the middle cerebral artery (MCA), as evidenced by cerebral magnetic resonance imaging [1]. Newborns with arterial stroke develop what is called cerebral palsy and exhibit symptoms including motor, cognitive, and/or behavioral disabilities and seizures. Seizures represent the clinical outcome that triggers assessment in neonates with stroke [2]. Perinatal stroke appears to be more frequent in boys, who have a worse outcome than girls, and ischemic injury appears to be more common in boys regardless of lesion type [3,4]. The bases for these sex differences are poorly understood, but sex differences in cell death [5], inflammatory responses, microglial activation [6], metabolic profile, and brain structure and plasticity [7] have been suggested to have a role [8].

The activation of resident microglial cells, alongside the infiltration of peripheral monocytes, represents a central inflammatory response to ischemic stroke leading to secondary injury cascade associated with neuronal death. The immune system plays a major role in determining the condition of the brain and the survival of patients following arterial stroke. Circulating monocytes differentiate into tissue macrophages and present common antigens and morphology with microglia. Microglia/macrophages are the main cells propagating inflammation not only in the core of the infarct but also to neighboring tissues. They are the major producers of inflammatory mediators, including cytokines and chemokines. Microglia/macrophages also play important roles in brain repair and regeneration [9]. The inflammatory microenvironment markedly influences the phenotype of microglia/macrophages, which may switch between inflammatory (M1) and protective (M2) phenotypes in response to specific activation cues [10]. We previously reported that permanent MCA occlusion (pMCAo) in postnatal 9-day-old (P9) mice induced microglia/macrophage activation with M1 and M2 phenotypes three days later [11], but we did not evaluate whether a differential effect may occur between both sexes. In the neonatal ischemic brain, M1 microglia secrete pro-inflammatory cytokines, such as COX-2, TNFα, and IL-1β, which exacerbate inflammation and tissue injury. M2 microglia/macrophages secrete best characterized markers such as Arginase-1 (Arg-1) and mannose receptor (CD-206) [11,12].

Sex-dependent differences in stroke (tandem pMCAo and ipsilateral common carotid artery (CCA)) vulnerability were demonstrated in adult C57BL/6 mice, with smaller infarct size in females (F) J and ByJ substrain mice than the corresponding males (M) [13]. Furthermore, F of the C57BL/6J strain sustained smaller infarcts than M after pMCAo. This could not be specifically related to the arterial blood pressure level or the collateral extent [14].

Sex diversity in preclinical studies was greatly encouraged. We recently reported that neonatal P9 C57BL/6J F mice subjected to pMCAo exhibited lower tissue loss than M three months after ischemia without any difference in behavioral deficits [12]. We here investigated, by using the same P9 mice stroke model, whether early sexual dimorphism may in part account for inflammatory responses, including M1 and M2 markers and immune infiltrated cells, to produce later differential cerebral tissue survival; this direction was chosen because new insights into sex differences in both microglia [15] and peripheral myeloid cells [16] following hypoxia–ischemia (HI) have been suggested these last years.

## 2. Results

### 2.1. No Sex Differences in Neuronal Loss and Lesion Size Three Days after Ischemia

**Figure 1:**
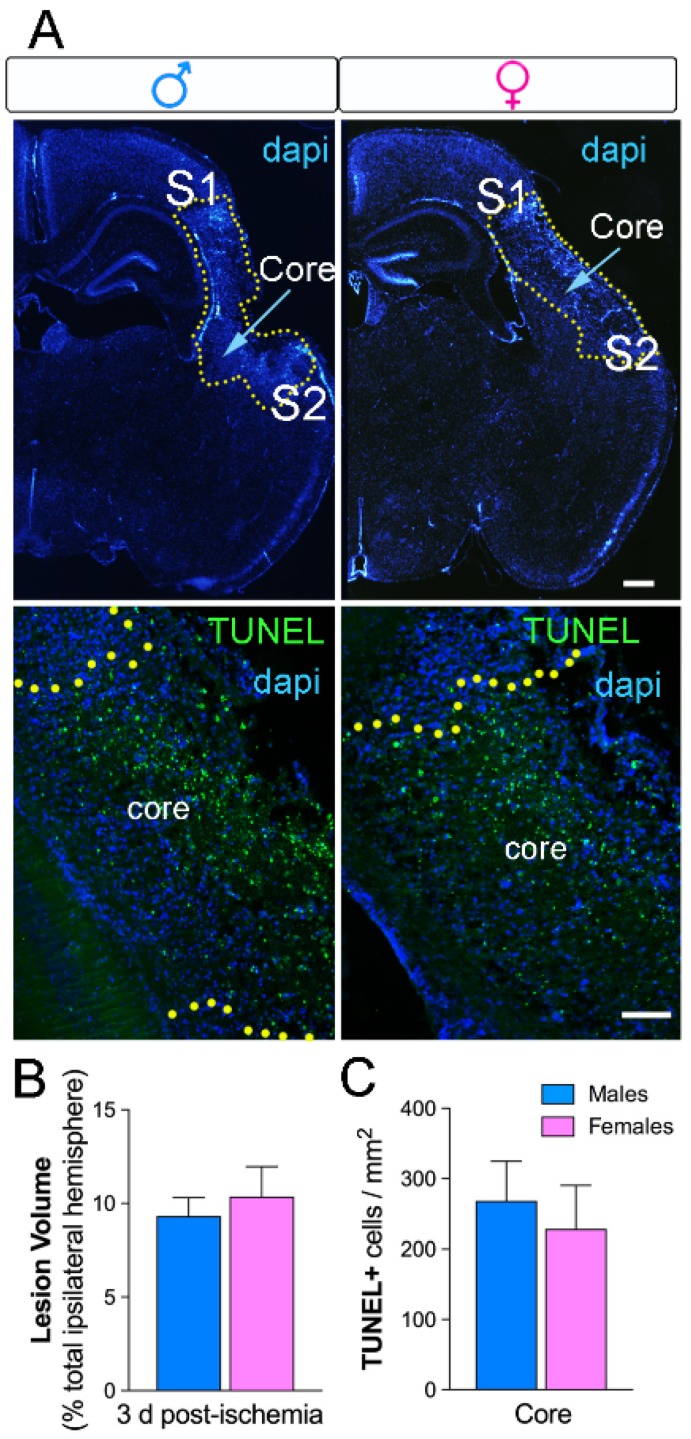
No differences in lesion volume and cell death in male and female ischemic mice. (**A**) The lesion was delineated (yellow dots) in 4′,6-diamidino-2-phenylindole (DAPI)-stained sections of the ipsilateral hemisphere after three days post-ischemia (upper images, scale bar = 100 µm). The primary somatosensory area (S1) and the secondary somatosensory area (S2) are indicated in the images and correspond with the peri-infarct region in the cortex. Representative images for TUNEL^+^ nuclei (green) and DAPI (blue) show the core region in the ischemic cortex (lower images, scale bar = 50 µm). (**B**) Lesion volumes in M (blue) and F (pink) mice at three days post-ischemia represented as the percentage of the total ipsilateral hemisphere. (**C**) No sex differences in the number of TUNEL^+^ nuclei, *n* = 5/group.

We examined ischemia-induced lesion size and neuronal cell death in the cerebral cortex. The percentage of lesion size after ischemia was 9.3 ± 0.9% of the total ipsilateral hemisphere in M and 10.3 ± 1.5% in F (Figure 1A,B; *n* = 5 per group). There were no sex-dependent differences in the injured volume, as previously reported. In the same way, we did not observe differences between M and F in the number of cells dying, with 268 ± 56 Terminal UTP nick-end labeling (TUNEL^+^)-nuclei per mm^2^ in M and 228 ± 62 TUNEL^+^-nuclei per mm^2^ in F, respectively (Figure 1A,C; *n* = 5 per group).

### 2.2. Sex Differences in Microglial Genes Three Days after Ischemia

**Figure 2:**
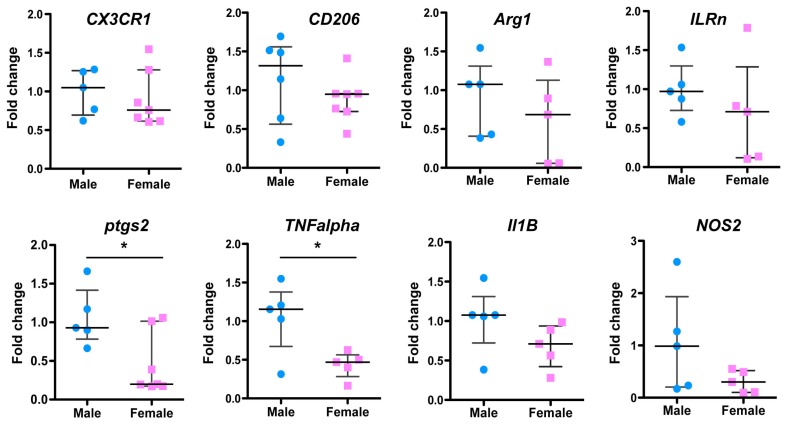
Sex differences in microglial gene expression three days after neonatal ischemia. Brains were analyzed for mRNA transcription after microglia magnetic cell sorting. Gene expression of M1- and M2-like markers in CD11b^+^ microglia from ischemic M (blue) and F (pink) mice using RT-qPCR. Each datum in M and F is expressed as a ratio against control referenced gene expression in CD11b^+^ microglia isolated from naive M and F mice. Data are reported as the median and 25th–75th percentiles, *n* = 5–7/group. * *p* < 0.05 M versus F.

We then analyzed the expression of microglial phenotypes (M1 and M2) on the microglial-enriched fractions obtained from M and F brain tissues three days after ischemia using RT-qPCR (Figure 2; *n* = 5–7 per group). No difference in the *CX3CR1* gene expression between both sexes was observed on CD11b^+^-microglia/macrophages. However, significantly lower (by half) gene expression was measured in F versus M for the two pro-inflammatory M1 markers Cox-2 (ptgs2, *p* < 0.05), and *TNFα* (*p* < 0.05), but not for the *Il-1β* and *NOS2* genes. Conversely, no difference was observed for the two anti-inflammatory M2 gene markers *CD206* and *Arg1*. 

### 2.3. Sex Differences in Microglial Phenotypes Three Days after Ischemia

**Figure 3:**
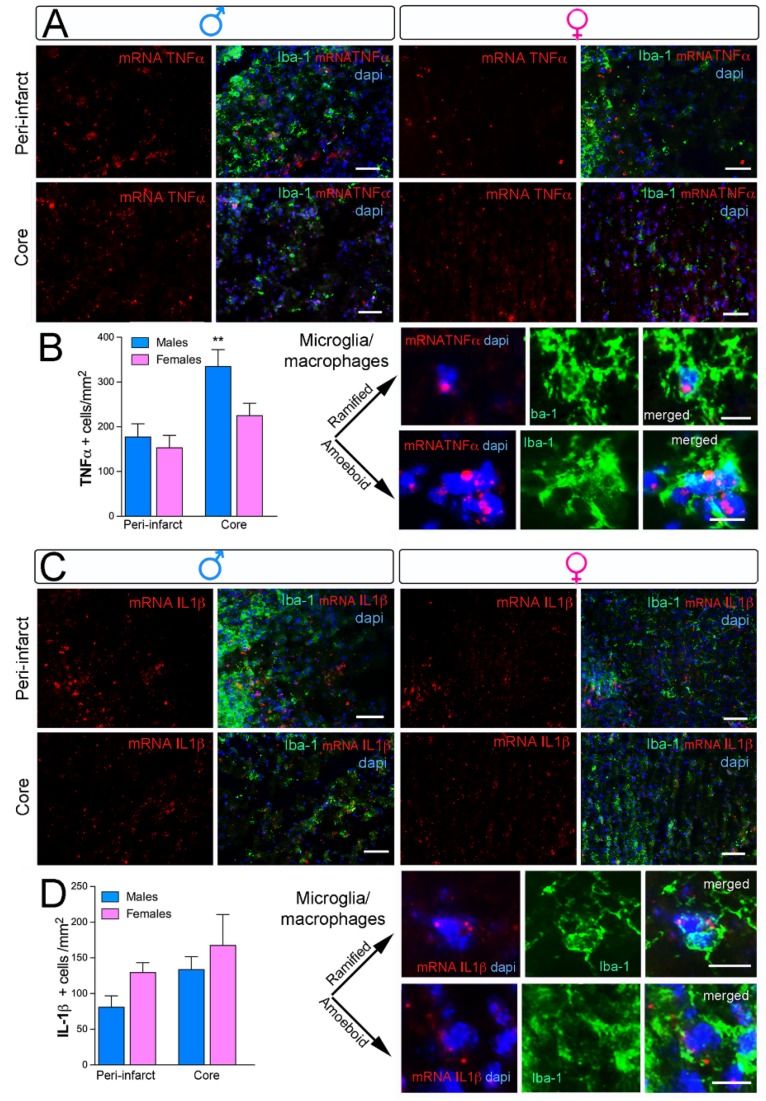
Sex-specific *TNFα* cytokine mRNA expression but not *Il-1β* after neonatal ischemia. (**A**,**C**) Representative images for *TNFα-* and *Il-1β*-positive cells (red), Iba-1 (microglia/macrophages, green), and DAPI (nuclei, blue) in the peri-infarct and core regions. Both *TNFα-* and *Il-1β*-positive cells were expressed in microglia/macrophage cells showing ramified or amoeboid morphology. Scale bars are 50 µm for the upper images and 20 µm for high-magnification images. (**B**) Increased mRNA *Il-1β*-positive cells in the core areas in M compared with F at three days post-injury. No significant differences in the peri-infarct region. (**D**) No significant differences in mRNA *Il-1β* density in M and F after injury, ** *p* < 0.01 (*n* = 5/group).

To further investigate this sex-dependent M1 marker, we quantified mRNA *TNFα* and *Il1-β* expression after ischemia using fluorescent in situ hybridization (FISH). In general, M had higher ipsilateral cortical levels of *TNFα* pro-inflammatory cytokine at three days post-ischemia compared with F (Figure 3A,B; *n* = 5 per group). We observed higher *TNFα* expression in the ipsilateral peri-infarct corresponding to the primary somatosensory cortex and the core of the lesion in M compared with F (Figure 3A). There was no detectable *TNFα* expression in the contralateral hemisphere (data not shown). FISH in combination with Iba-1 antibody showed that the majority of *TNFα* colocalized with microglia cells in the ipsilateral peri-infarct but not in the cortical core. This suggests that *TNFα* is not only produced by microglia with ramified morphology but also by microglia/macrophages with amoeboid morphology (Figure 3A, high-magnification images). Besides this, another proinflammatory cytokine, *Il-1β*, was analyzed in the core and peri-infarct of M and F ischemic mice. We did not observe any sex-dependent differences in the expression of this cytokine at three days post-ischemia (Figure 3C,D; *n* = 5 per group). *Il-1β*-positive cells colocalized with microglia/macrophage cells displaying ramified and amoeboid morphology in the peri-infarct area (Figure 3C, high-magnification images).

### 2.4. Higher Myeloid Infiltrate in Males Versus Females Three Days after Ischemia

**Figure 4:**
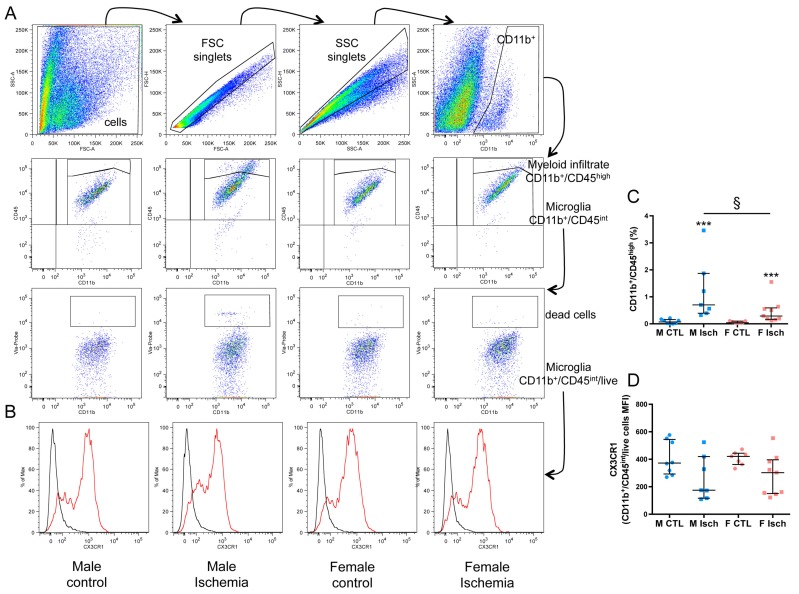
FACS analysis of myeloid infiltrate and microglial expression of CX3CR1. (**A**) Gating strategy for microglia (CD11b^+^/CD45 ^int^/live) and myeloid infiltrate (CD11b^+^/CD45 ^high^): cells and singlets were selected based on morphological parameters, then CD45 ^int^ and CD45 ^high^ cells were gated in CD11b^+^ cells, and dead cells were excluded in CD11b^+^/CD45 ^int^ cells. Forward scatter (FSC); (**B**) CX3CR1 expression was measured in microglia (CX3CR1 signal in red line; control isotype in black line). (**C**) Percentage of myeloid infiltrate in whole brain (% in side-scatter (SSC) singlets) in M (blue) and F (pink) control (circles) and ischemia (squares). Control (CTL); ischemia (Isch). (**D**) Mean fluorescence intensity (MFI) of CX3CR1 in microglia in M (blue) and F (pink) control (circles) and ischemia (squares). Data are reported as median and 25th–75th percentiles (*n* = 7 to 9/group). *** *p* < 0.001, non-parametric Mann–Whitney test vs. control, ^§^ *p* < 0.05, non-parametric Mann–Whitney test vs. M.

Inflammatory responses involve the activation of resident microglia and infiltration of peripheral myeloid cells (polymorphonuclear neutrophils (PMNs) and monocytes) in the ischemic brain. As shown with flow cytometry, the percentage of myeloid cells (gated as CD11b^+^/CD45 ^high^, Table 1) in M (0.7% (0.4–2)) was significantly higher than that in F (0.3% (0.2–0.6)) (*p* = 0.0418) three days after ischemia (Figure 4A,C). In addition, the CD11b mean fluorescence intensity (MFI) was increased in ischemic F (*p* = 0.0079) as compared to control F. No difference in the percentage of total resident microglia (gated as CD11b^+^/CD45 ^int^) was seen between control and ischemic tissue whatever the sex (Figure 4B and Table 1). In control groups, CD11b^+^-microglia were ~60% CX3CR1 in M and ~69% in F (*p* = 0.0541). We then examined CX3CR1 expression by analyzing the percentage of positive cells and MFI in both M and F ischemic mice. Although a reduced mean number of CX3CR1^+^ microglia was found in M after ischemia as compared to F, this trend did not reach significance. No significant difference was observed in MFI three days after ischemia (Figure 4B,D and Table 1). Lymphocytes have been reported to contribute to neonatal ischemia [17] and HI [18]. Using a CD3 antibody against pan-T lymphocytes, numerous CD3^+^ cells were found in the ischemic core and leptomeningeal membranes in the same density in M and F (Figure S2).

### 2.5. Increased Microglia Immunoreactivity in Males Versus Females Three Days after Ischemia

**Figure 5:**
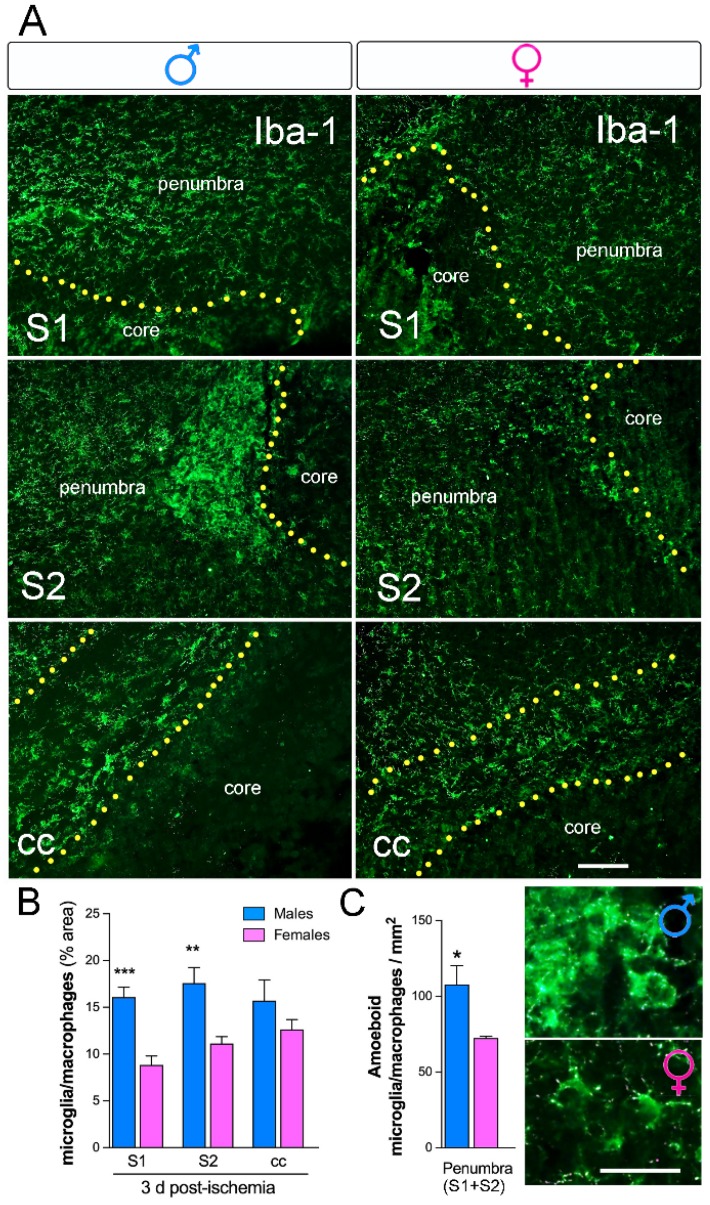
Sex-dependent differences in microglia/macrophage density after neonatal ischemia. (**A**) Representative images of Iba-1 immunohistochemical staining (microglia/macrophages) show an increase in M ischemic brains compared to the F in the primary (S1) and secondary (S2) somatosensory area and in the cortical core. (**B**) Sex differences observed in the cortical S1 and S2 regions with higher Iba-1 staining in M compared to F at three days post-ischemia. No differences were observed in the corpus callosum (cc) region (**C**). Iba-1-positive cells classified according to amoeboid/hypertrophy microglia/macrophage morphology in the peri-infarct (S1 and S2 areas). M mice showed a higher increase in amoeboid cells than did F mice. Representative images of Iba-1 staining show an increase in cells with amoeboid morphology in M compared to F. *** *p* < 0.001, ** *p* < 0.01, * *p* < 0.05, *n* = 5/group.

We analyzed the microglia/macrophage activation in several brain regions using Iba-1 staining. We observed an increase in the area occupied by Iba-1 in M versus F (Figure 5A,B; *n* = 5 per group) in the peri-infarct corresponding to the primary (S1) and secondary (S2) somatosensorial cortex. No significant changes in the area occupied by Iba-1^+^-cells in the corpus callosum (cc) were observed. Measurements of the percentage of the total area occupied by Iba-1^+^ cells were almost twofold higher in M than in F ischemic brains in S1 (Figure 5B). In the peri-ischemic region a higher density of Iba-1^+^ cells with amoeboid morphology was observed in M compared to F (Figure 5C, high-magnification images; *n* = 5 per group).

## 3. Discussion

Only a limited number of studies have investigated the effect of sex on microglial activation after ischemic stroke in the developing brain, and it is now quite recognized that microglia “go sexist” [19]. In this study, we examined sex-mediated inflammatory and immune responses after neonatal stroke. We found that M show more peripheral myeloid cell infiltration and pro-inflammatory markers than F, although both sexes exhibit equivalent tissue damage three days after ischemia. These early brain responses to stroke could in part explain why females display smaller infarcts in adulthood, as previously reported [12].

Male sex is a well-established epidemiological risk factor for poor neurodevelopmental outcome after perinatal brain injury, and male infants experience more intrapartum complications and/or worse pregnancy [20,21,22]. The mechanisms responsible for this sex difference are not yet fully understood. Estradiol is at its highest level during the perinatal period; its levels in the cortex are similar in M and F, but its levels in the hypothalamus are higher in M compared to F rats. Microglia express both isoforms of the estrogen receptor (α and β) [23,24], and estrogens inhibit microglial apoptotic signaling and downregulate various cytokines [24], suggesting that steroid hormones may regulate microglial activation in F. Steroid hormones may also account for the differences between control M and F for some microglial genes. Numerous studies point to a protective role of estrogen in F in response to an injury stimulus. However, in neonates and primary microglial cultures, microglial cells differ between M and F when the levels of estrogen, progesterone, and testosterone are basal, suggesting that alternative signaling pathways are responsible for sex differentiation under neuroinflammatory conditions [8]. Indeed, M and F C57BL/6 mice respond differently to HI, but M and F have equivalent circulating hormones [15,25].

Inflammation following neonatal stroke was evidenced not only by microglial activation but also by peripheral immune responses with leukocyte, macrophage, and lymphocyte recruitment [14,26]. One day after transient MCAo in the P7 rat (sex was not determined), monocyte infiltration was low, and the majority of macrophages in acutely injured regions were microglia [27]. Two to three times more CD11b^+^/CD45 ^high^ infiltrating cells were measured in M compared to F in our study. Similar data were reported on Day 3 (but not on Day 1) after HI (Rice–Vannucci model, RVM) in the C57BL/6 mouse, mainly including a higher density of monocyte and lymphocyte recruitment in M compared to F [15]. Using the same mouse strain, it was reported that peripheral myeloid cells contribute to brain damage after HI in P9 mice and that myeloid cell depletion reduced brain damage in M and not in F [22]. However, in this study, myeloid cells were significantly expressed at 1 and 7 days after HI [16]. Together, these data suggest that peripheral immune responses may depend on the rodent species and strains used, the age of the animals (from P5 to 7 in rats and P9 to P10 in mouse), and the model selected (ischemia versus HI). In the same RVM model, infiltration of peripheral leukocytes was still detected 14 [16] and 30 days [25] after injury. Stroke induces blood–brain barrier (BBB) permeabilization, leading to the infiltration of leukocytes which release cytokines and chemokines and then amplify inflammatory responses over days and weeks. Infiltrated monocytes were able to clear debris of dying cells at 72 h after ischemia in C57BL/6J mice [28]. Monocytes firstly express pro-inflammatory markers within 3 days, then after 7 days, half of them express anti-inflammatory markers; when monocyte recruitment is blocked during the first week after ischemia, long-term behavioral recovery is abolished in adult ischemic C57BL/6J mice [29].

No differences in the number of microglia were found in the fetal brain of M and F before birth. However, sex differences begin after birth in both number and morphology (and gene expression) with more microglia in M compared to F at postnatal Day 4 in different regions of the brain, including the cortex, hippocampus, and amygdala [6,30]. Microglial activation and aggregation are pathological hallmarks for stroke in human infants [31] and neonatal mice [10]. In basal conditions, the activation of microglia is characterized by high expression of the chemokine receptor CX3CR1 [32] and/or MHC class II [33]. Three days after ischemia, our qPCR and FACS analyses showed the same amount of CX3CR1 (a regulator of phagocytic clearance [34]) both in M and F, although with a trend to higher CX3CR1 percentage in F not reaching significance. However, immunostaining with Iba-1, a microglial/macrophage-specific marker, demonstrated that M had significantly more Iba-1^+^ cells than F both in the core and peri-infarct regions (maybe some Iba-1+ microglia did not express CX3CR1) and that these cells had an amoeboid morphology, in agreement with the above data showing more infiltrating myeloid cells including monocytes in M versus F. Loss of CX3CR1 reduced monocyte recruitment and expansion of microglia, leading to reduced inflammation after MCAo in adult ischemic M mice (F mice were not studied) [35]. CD11b expression measured by FACS was significantly increased in F after ischemia. A trend of CD11b increase was also observed in males, but without reaching statistical significance, whereas myeloid cell infiltration was more pronounced in these males. CD11b is a classical activation marker in immune myeloid cells and microglia [36]. In microglia, CD11b also plays a role as an “eat me” signal receptor during synaptic pruning [37]. There might be an association between the variations of microglia CD11b expression and myeloid cell infiltration in the brain, as shown by Perego et al. [38]. In adult M mice, they observed that the depletion of peripheral myeloid cells inhibited their brain infiltration 24 h after ischemia and was associated with increased CD11b expression in the ischemic area. They proposed that myeloid infiltration might regulate the local inflammatory response in an M2 protective way. The same association might already exist in neonates, with less infiltration/higher local CD11b expression/less tissue injury in females, and more infiltration/limited CD11b increase/more tissue injury in males. In another model with earlier assessment, only M adult C57BL/6J mice exhibited an increase in CD11b immunoreactivity in proximal and distal cortical regions immediately after a 60 min pMCAo, whereas in basal conditions, CD11b staining was prevalent in the F when compared to M tissues [39]. These observations suggest both sex- and time-dependent regulation, which requires further investigations to be fully understood. The additional role of CD11b as an “eat me” receptor could, in our model, lead to greater pruning capacity of female than male microglia, but our results do not allow us to conclude about the positive or detrimental effects of this difference. 

According to the environment and the production of cytokines, microglia can adopt an inflammatory/cytotoxic phenotype (M1-like) and/or an immunomodulatory/repairing phenotype (M2-like) [40], although the question of whether and how these changes occur can vary according to model (in vitro vs. in vivo) and brain developmental stage (immature vs. mature) [41]. It was found that microglia are both M1 and M2 during the acute stage of neuroinflammation induced by a stroke in adults [42,43]. Our data show that F ischemic brains respond to ischemia with a significant decrease in the M1 phenotype (Cox-2 and TNFα gene expression) at three days after injury when compared to M, whereas no difference was detected in markers of the M2 phenotype, suggesting that the pro-inflammatory response is delayed in neonatal ischemic brains, probably because infarct volume increases with time [10,12]. Interestingly, *TNFα* gene expression was more strongly detected in M in the core and in both the distal and proximal peri-infarct areas. The expression of two M2 phenotype markers (CD-206 and Arg-1) was not different between M and F brains. MRC-1 (CD-206 gene) and Arg-1 were previously reported to be present three days after ischemia in perivascular microglial/macrophages in leptomeninges from where they entered the neocortex along the penetrating arterioles in the peri-infarct [10], suggesting that these cells may be absent from our CD11b^+^-microglia population obtained after brain perfusion and dissociation.

A limitation of the present study is that only one time-point of recovery was evaluated, and several questions about the role of CX3CR1 and CD11b markers in both M and F brains need further analyses both in basal conditions and at different time points after ischemia. Altogether, both central and peripheral immune responses showed a differential pattern in M and F as early as three days after neonatal stroke, though with equivalent primary brain injury (infarct volume). These different patterns may have a more significant impact on secondary neuronal damage because the final lesion size is reduced in F in adulthood [12].

## 4. Materials and Methods

### 4.1. Ethics Statement

All care and experiments were carried out under the ethical approvals stipulated by the French Department of Agriculture (APAFiS#15396) in accordance with the European Communities Council Directive of 22 September 2010 (2010/63/EEC) on the protection of animals for experimental use and conformed to the Guide for the Care and Use of Laboratory Animals published by the U.S. National Institutes of Health (publication 85–23, revised 1996). Sample sizes were determined by power analysis according to our previous report [11].

### 4.2. Neonatal Ischemia

Twelve litters of C57BL6/J mice (wild type) were purchased from Janvier Labs (Le Genest-St Isle, France), and animals were visually sexed. Permanent proximal middle cerebral artery (MCA) occlusion (pMCAo) was carried out by electrocoagulation [11] under isoflurane anesthesia (Vetflurane, Virbac, Switzerland) in 30% O_2_ and 70% N_2_O in P9 mice (*n* = 39; 17 M and 22 F). Mice were sacrificed 3 days after ischemia (P12), and all animals presented a pale cortical lesion on the ipsilateral hemisphere. Age-matched control (naive) P12 mice (*n* = 25; 13 M and 12 F) were used when necessary. All animals survived until P12 and were randomized for each group; all analyses were performed blinded to surgical and sex conditions (Figure S1). For tissue preparations, see the Supplementary Material.

### 4.3. Brain Tissue Dissociation and Magnetic-Activated Cell Sorting (MACS)

Brains were collected for cell dissociation using a Neural Tissue Dissociation Kit (Miltenyi Biotec., Bergisch Gladbach, Germany), and microglial cells were then enriched using a magnetic-bead-coupled antibody (anti-CD11b, Miltenyi Biotec, dilution 1:10) extraction technique (MACS™) (Miltenyi Biotec.) according to the manufacturer’s protocol using all recommended reagents and equipment [44]. 

### 4.4. Real-Time Quantitative Reverse Transcriptase Polymerase Chain Reaction

Total RNA was extracted from purified CD11b-positive cells (see above) of each M and/or F control and ischemic P12 animal as previously described [44]. Primer3 plus software was used to design primers (for sequences, refer to Supplementary Table S1). The expression of genes of interest was calculated relative to the expression of the reference ribosomal protein L13 (Rpl13a) gene. Then, gene expressions in ischemic M and F samples were calculated relative the averaged corresponding control value (M and/or F) and expressed as fold changes (log_2_). Analyses were performed using Bio-Rad CFX Manager 3.0 (Bio-Rad, Hercules, CA, USA).

### 4.5. Flow Cytometry

The brain tissues (*n* = 6–9 each group) were dissociated using a Neural Tissue Dissociation Kit (Miltenyi Biotec.; see above). After counting and resuspension at 10 × 10^6^ cells/mL, cells were incubated with fluorophore-conjugated antibodies against mouse CD45, CD11b (both Miltenyi Biotec GmbH), CX3CR1 (Sony Biotechnology, San Jose, CA, USA), or their corresponding control isotypes (Miltenyi Biotec GmbH and Sony Biotechnology) and BD Via-Probe (BD Biosciences, Le Pont De Claix, France) at concentrations recommended by the manufacturers or calculated after titration. FACS analysis was done within 24 h. After doublets exclusion based on morphological parameters, the gating strategy selected microglia as CD11b+/CD45 ^int^/live cells and myeloid cells (including polymorphonuclear neutrophils, monocytes, and macrophages) as CD11b+/CD45 ^high^ cells. Viability rate was not measured in the myeloid cells because the number of events was frequently too small.

### 4.6. Immunohistochemical Techniques

Coronal brain sections were incubated with a polyclonal antibody goat anti-rabbit Iba-1 (Wako, Chemicals, Richmond, VA, Canada, 1:500) for microglia/macrophages overnight at 4 °C and revealed with Alexa Fluor 488-conjugated goat anti-rabbit IgG (1:1000, Invitrogen, Carlsbad, CA, USA). Sections were then counterstained with DAPI (1:50,000, Sigma-Aldrich, St. Louis, MO, USA). 

### 4.7. Fluorescent In Situ Hybridization Combined with Immunofluorescence Staining

Fluorescent in situ hybridization (FISH) was performed as per the manufacturer’s instructions using an RNAscope^®^ Technology 2.0 Red Fluorescent kit (Advanced Cell Diagnostics (ACD), Hayward, CA, USA) as we have previously described [45]. Brain tissue sections were dehydrated before hybridization. Sections were then incubated at 40 °C for 2 h with the target probes for mouse: Mus musculus interleukin 1 beta (IL-1β) mRNA (accession number NM_008361.3, target region 2–950) and Mus musculus Tumor necrosis factor alpha (TNFα) mRNA (accession number NM_013693.2, target region 41–1587). A dapB probe targeting a bacterial gene (coding for dihydrodipicolinate reductase) was used as a negative control and Ppib (Mus musculus peptidylprolyl isomerase B mRNA; accession number NM_011149.2, target region 98–856) was used as a positive control. Positive hybridization consisted of a punctate signal representing a single mRNA target molecule; the color label was assigned to FAR RED (excitation 647 nm; emission 690  ±  10 nm). 

### 4.8. Densitometry and Quantitative Analysis of Immunofluorescence and FISH

The number of mRNA-positive cells colocalized with DAPI nuclei was manually counted for IL-1β and TNFα. The negative probe used as a control did not contain any stained cells. A total of 15–20 counting frames were assessed per animal, and cells were evaluated for the presence of a labeled nucleus and expected cellular morphology as previously described [46]. For proportional area measurements, the magnitude of the individual reaction for microglial and macrophages (Iba-1^+^ cells) was reported as the proportional area of tissue occupied by immunohistochemically stained cellular profiles within a defined target area. Proportional area measurements do not necessarily reflect changes in actual cell numbers. Images were transferred to ImageJ64 software 1.8.0 (NIH Bethesda, MD, USA) for inversion, thresholding, and densitometric analysis. The thresholding function is used to set a black and white threshold corresponding to the imaged field, with the averaged background subtracted out. Once a threshold is set, the “Analyze Particles” function can be used to sum up the total area of positive staining and to calculate the fraction of the total area that is positive for the stain. Data are shown as the percentage of Iba-1-positive immunoreactivity per the total area occupied on the field studied. 

### 4.9. Cell Death Assay

Brain sections (5 M and 5 F) were processed for DNA strand breaks (TUNEL assay labeling of fragmented DNA) using a Fluorescence In Situ Cell Death Detection kit (Roche Diagnostic, Indianapolis, IN) according to the manufacturer’s instructions. 

### 4.10. Statistical Analysis

Data (TUNEL^+^-nuclei, lesion volume, and cell counts) are expressed as the mean ± SD of *n* observations. FACS and qPCR data results were expressed as individual values with the median and the interquartile range (IQR). All figures and statistical analyses were created with GraphPad Prism 7.0 (GraphPad Software, San Diego, CA, USA). The Kruskal–Wallis test was used to compare data between two groups (M vs. F in lesion volume, TUNEL and cell counts, and qPCR data). Differences between the four groups were tested using one-way ANOVA. When statistical significance was found, two-by-two intergroup differences were tested with the Mann–Whitney test. 

## 5. Conclusions

The present study demonstrated sex differences in gene expression and peripheral infiltrated cells after stroke in the neonatal brain at 72 h, which could in part explain some differences in long-term outcomes. These data also suggest the need for developing sex-specific therapy strategies in neonatal (and maybe pediatric) intensive care units. Indeed, the convergence of M sex and early (during brain development) inflammation represents a greater risk factor for further neuropsychiatric disorders [47]. 

## Figures and Tables

**Table 1:** FACS analysis of microglia and myeloid cell infiltrate in mouse brains.

Microglial Cells	M CTL	M Isch	F CTL	F Isch	*p*
					Kruskal–Wallis
					Test
% CD11b^+^/CD45 ^int^ (microglia)	9.3	9.5	7	7.7	0.2588
	(8.7–11.1)	(7.6–9.7)	(6.6–8.4)	(5.3–10.1)	
(*n*)	(8)	(7)	(7)	(9)	
% Dead cells in CD11b^+^/CD45 ^int^	1.3	2.6	0.8	2.4	0.014
	(0.7–2.3)	(2.0–3.3)	(0.6–1.0)	(1.5–2.7)	
(*n*)	(5)	(4)	(4)	(5)	
				* *p* = 0.0159	
% CD11b^+^/CD45 ^high^ (myeloid infiltrate)	0.07	0.7	0.04	0.3	<0.0001
	(0.0005–0.2)	(0.4–2)	(0.03–0.1)	(0.2–0.6)	
(*n*)	(8)	(7)	(7)	(9)	
		* *p* = 0.0003		* *p* = 0.0002	
				§ *p* = 0.0418	
CD11b Mean Fluorescence Intensity (MFI)	3123	4036	3195	4548	0.0082
	(3006–3424)	(3142–4527)	(2995–3506)	(3715–4896)	
(*n*)	(6)	(6)	(7)	(9)	
				* *p* = 0.0079	
% CX_3_CR1+ cells in CD11b^+^/CD45 ^int^	59.6	46.2	68.7	63.9	0.0359
	(52.1–65.9)	(33.4–61.2)	(63.4–72.3)	(42.2–69 0)	
(*n*)	(8)	(7)	(7)	(9)	
			§ *p* = 0.0541		
CX_3_CR1 MFI in CD11b^+^/CD45 ^int^	373	175	421	302	0.1004
	(293–544)	(116–419)	(362–444)	(150–397)	
(*n*)	(8)	(7)	(7)	(9)	

Data are reported as median (25th–75th percentiles). Global comparison between the four groups—male control (M CTL), M with permanent middle cerebral artery occlusion (pMCAo) (M Isch), female control (F CTL), and F with pMCAo (F Isch)—was made by non-parametric Kruskal–Wallis test. Two-by-two comparisons were made by non-parametric Mann–Whitney test: * *p* < 0.05 vs. controls, § *p* < 0.05 vs. M. For the CD11b^+^/CD45 ^int^ (microglia) population, 4328 (2844–7964); 4229 (3673–5544); 3079 (2941–3781); and 3677 (2552–4425) cells were analyzed in M CTL, M Isch, F CTL, and F Isch, respectively. Cell numbers for myeloid infiltrate (CD11b^+^/CD45 ^high^) were 26 (13–135); 568 (172–841); 17 (13–46); and 137 (73–278), respectively. (*n*)= number of mice analysed

## Supplementary Materials

Supplementary materials can be found at https://www.mdpi.com/1422-0067/20/15/3809/s1.
