# Supplementary material for: Early Sex Differences in the Immune-Inflammatory Responses to Neonatal Ischemic Stroke
Source: Int J Mol Sci. 2019 Aug 4;20(15):3809. doi: 10.3390/ijms20153809 (PMC6695584; doi:10.3390/ijms20153809)
Supplement: Supplementary file 1 [file ijms-20-03809-s001.pdf]

Supplementary Material

Early sex differences in the immune-inflammatory responses to neonatal ischemic stroke

Sonia Villapol, Valérie Faivre, Pooja Joshi, Raffaella Moretti, Valérie C Besson and Christiane Charriaut-Marlangue

Materials and Methods

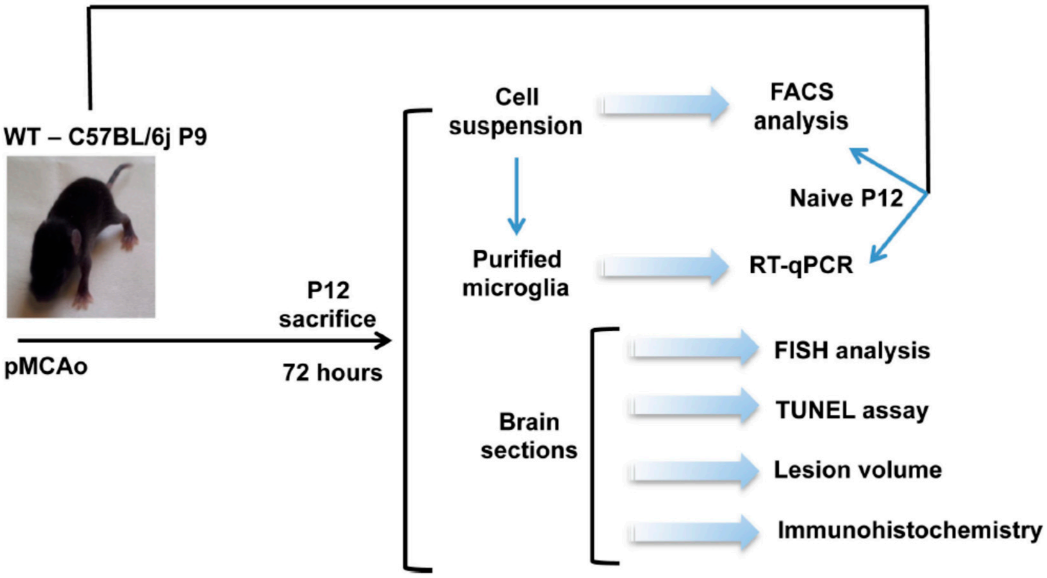

Figure S1. Outline of the experimental procedure in P9 C57Bl/6j mice subjected to pMCAo.

Tissue harvesting.

For cell sorting, mice were euthanized, transcardially perfused with cold sterile saline buffer, and the brains were harvested. The brainstem, cerebellum, and olfactory bulbs were removed. The brain was then divided along the interhemispheric fissure, and the ipsilateral (left) side used for molecular biology experiments. For immunohistochemical analysis, mice were anesthetized then transcardially perfused with phosphate-buffered saline (PBS) and decapitated. Brains were removed, fixed in 4% formol (Sigma-Aldrich) overnight, and then embedded in paraffin and cut into 16 µm-thick sections.

Brain tissue dissociation and magnetic-activated cell sorting (MACS).

The purity of MACSed CD11b$^+$ fractions was validated with RT-qPCR of the positive and negative CD11b cell fractions. We used RT-qPCR for glial fibrillary acid protein (Gfap), neuronal nuclear antigen (NeuN), Myelin basic protein (Mbp) and Integrin Alpha M (Itgam) gene that encode CD11b, and confirmed that NeuN, Gfap and Mbp mRNA expression levels were extremely low compare to Itgam mRNA expression [44].

Immunohistochemistry

Dissected brains were fixed by immersion in formalin-free fixative (RCL2, Alphelys, Plaisir, France) and embedded in paraffin. Coronal 16-µm thick brain sections (from P12 ischemic animals, n=5/group) at the MCA and hippocampal level were cut from the blocks

with a Leica microtome. Sections were washed three times with PBS and incubated with blocking buffer (3% normal goat serum (NGS) in PBS) and 0.3% Triton (PBST) for 2 h at room temperature. Brain sections were incubated with a polyclonal antibody goat anti-rabbit Iba-1 (Wako, Chemicals, Richmond, VA, 1:500) for microglia/macrophages overnight at 4°C. After three 5 min washes in PBS, brain sections were incubated with Alexa Fluor 488-conjugated goat anti-rabbit IgG (1:1,000, Invitrogen, Carlsbad, CA) for 2 h at room temperature. Sections were rinsed with PBS three times and washed in PBS with DAPI (1:50,000, Sigma-Aldrich, St. Louis, MO) solution for counterstained nuclei. Sections were washed three times with distilled water and coverslipped with Fluoro-Gel with Tris Buffer mounting medium (Electron Microscopy Sciences, Hatfield, PA).

Fluorescent in situ hybridization combined with immunofluorescence staining.

Fluorescent in situ hybridization (FISH) was performed as per the manufacturer's instructions using RNAscope® Technology 2.0 Red Fluorescent kit (Advanced Cell Diagnostics (ACD), Hayward, CA) as we have previously described [47]. Brain tissue sections were dehydrated by 50%, 70%, and 100% ethanol gradually for 5 min; boiled during 10 min with pretreatment 2 solution (citrate buffer), then they were incubated with pretreatment 3 solution (protease buffer) for 30 min before hybridization. Sections were then incubated at 40°C for 2 h with the target probes for mouse: *Mus musculus interleukin 1 beta* (IL-1β) mRNA, (accession number NM_008361.3, target region 2-950) and *Mus musculus Tumor necrosis factor alpha* (TNFα) mRNA, (accession number NM_013693.2, target region 41-1587). A dapB probe targeting a bacterial gene was used as a negative control and Ppib (Mus musculus peptidylprolyl isomerase B mRNA; accession number NM_011149.2, target region 98-856) was used as a positive control. Positive hybridization consisted of a punctate signal representing a single mRNA target molecule, the color label was assigned to either FAR RED (Excitation 647 nm; Emission 690 ± 10 nm). After FISH, slides were washed three times with PBS and blocking with PBST and 5% normal goat serum for 1h. Immunofluorescence for microglia/ macrophages was performed as was described previously.

Flow cytometry.

The brain tissues (n=6-9 each group) were dissociated after Neural Tissue Dissociation Kit (Miltenyi Biotec.; see above). The cells were then washed and re-suspended in FACs buffer (Dulbeccos's Phosphate Buffer Saline (Gibco Life Technologies, Paisly, UK), 2mM EDTA (Sigma Aldrich, Saint Louis, MO, USA), 0,5% Bovine Serum Albumin (Miltenyi Biotec GmbH, Bergisch Gladbach, Germany). After counting and resuspension at 10.10_6 cells/ml, cells were incubated with fluorophore-conjugated antibodies against mouse CD45, CD11b (both Miltenyi Biotec GmbH), CX3CR1 (Sony Biotechnology, San Jose, CA, USA) or their corresponding control isotypes (Miltenyi Biotec GmbH and Sony Biotechnology), and BD Via-Probe (BD Biosciences, Le Pont De Claix, France) at concentrations recommended by the manufacturers or calculated after titration. Cells were washed twice with FACs buffer, fixed with 1% paraformaldehyde (PFA, Sigma Aldrich) and FACs analysis was done within 24 h. After doublets exclusion based on morphological parameters gating strategy selected microglia as CD11b+/CD45int/live cells and myeloid cells (including polymorphonuclear neutrophils, monocytes and macrophages) as CD11b+/CD45high cells.

Viability rate was not measured in the myeloid cells because the number of events was frequently too small.

Real-time quantitative reverse transcriptase polymerase chain reaction.

Total RNA was extracted from purified CD11b-positive cells (see above) of each M and/or F control and ischemic P12 animal, using nucleospin RNA Plus XS kit (Macherey- Nagel, Hoerdt, France). Four hundred ng RNA was subjected to reverse transcription using the iScriptTM cDNA synthesis kit (Bio-Rad, France). A 2-step (5 s of denaturation at 95°C and 10 s of annealing at 60°C) RT-qPCR program was performed in triplicate for each sample using SYBR Green Super-Real-time mix (Bio-Rad). Amplification specificity was assessed with a melting curve analysis. Primer3 plus software was used to design primers (referred sequences in Table S1). The expression of genes of interest was calculated relative to the expression of the reference ribosomal protein L13 (Rpl13a) gene. Then, gene expressions in ischemic M and F samples were calculated relative the averaged corresponding control value (M and/or F) and expressed as fold change (Log2). Analyses were performed using Bio-Rad CFX Manager 3.0 (Bio-Rad, Hercules, CA).

***Densitometry and quantitative analysis of immunofluorescence and FISH*.**

Investigators blinded to the experimental conditions quantified FISH signal in three fields (x10, 514.560 mm_2) within the primary and secondary somatosensory cortex of four noncontiguous slices of the ischemic cortex (or cortical layer III-IV). The number of mRNA-positive cells that colocalized with DAPI nuclei was manually counted for IL-1β and TNFα. The negative probe used, as a control did not contain any stained cells. A total of 15–20 counting frames were assessed per animal, and cells were evaluated for the presence of a labeled nucleus and expected cellular morphology, as was previously described [46]. For proportional area measurements, the magnitude of the individual reaction for microglial and macrophages (Iba-1_+ cells) was reported as the proportional area of tissue occupied by immunohistochemical stained cellular profiles within a defined target area. Proportional area measurements do not necessarily reflect changes in actual cell numbers. Images were transferred to ImageJ64 software (NIH Bethesda, MD, USA), for inversion, thresholding, and densitometric analysis. The thresholding function is used to set a black and white threshold corresponding to the imaged field, with the averaged background subtracted out. Once a threshold is set, the "Analyze Particles" function can be used to sum up the total area of positive staining, and to calculate the fraction of the total area that is positive for the stain. Data are shown as the percentage of Iba-1 positive immunoreactivity per the total area that occupied on the field studied. The images were taken using Nikon microscope (Eclipse Ni- U), pco.edge 4.2 Scmos camera, analyzed and NIS-Element. Images were cropped and resized (if necessary) using Adobe Photoshop CC 2018; any changes to brightness and contrast settings were applied equally to the entire image, and to all corresponding images within the same dataset.

Cell death and infarct volume

Brain sections (5 M and 5 F) were processed for DNA strand breaks (TUNEL assay labeling of fragmented DNA) using the Fluorescence In situ Cell Death Detection kit (Roche, IL), according to the manufacturer´s instructions. TUNEL-positive nuclei were counted in cortical regions in the three to five coronal sections (x20) for each M and F

mouse. The lesion volume on cresyl violet-stained sections (n=6 sections; 500 micrometers between 2 sections) was calculated by multiplying the sum of the lesion areas by the distance between sections. Lesion percentage was calculated by dividing each lesion volume by the total contralateral hemisphere volume.

Table 1. Primer sequences.

mNOS2-F	CCC TTC AAT GGT TGG TAC ATG G
mNOS2-R	ACA TTG ATC TCC GTG ACA GCC
mPtgs2-F	TCA TTC ACC AGA CAG ATT GCT
mPtgs2-R	AAG CGT TTG CGG TAC TCA TT
mCd206-F	CTT CGG GCC TTT GGA ATA AT
mCd206-R	TAG AAG AGC CCT TGG GTT GA
mArg1-F	GTG AAG AAC CCA CGG TCT GT
mArg1-R	GCC AGA GAT GCT TCC AAC TG
mIl1Rn-F	TTG TGC CAA GTC TGG AGA TG
mIl1rn-R	TTC TCA GAG CGG ATG AAG GT
mTNFa_F	GCC TCT TCT CAT TCC TGC TT
mTNa_R	AGG GTC TGG GCC ATA GAA CT
mCX3CR1-WT-F	GTCTTCACGTTCGGTCTGGT
mCX3CR1-common-R	CCCAGACACTCGTTGTCCTT
mIL1b-F	GGGCCTCAAAGGAAAGATTC
mILl1b-R	TCTTCTTTGGGTATTGCTTGG

Results

Immunohistochemistry for CD3.

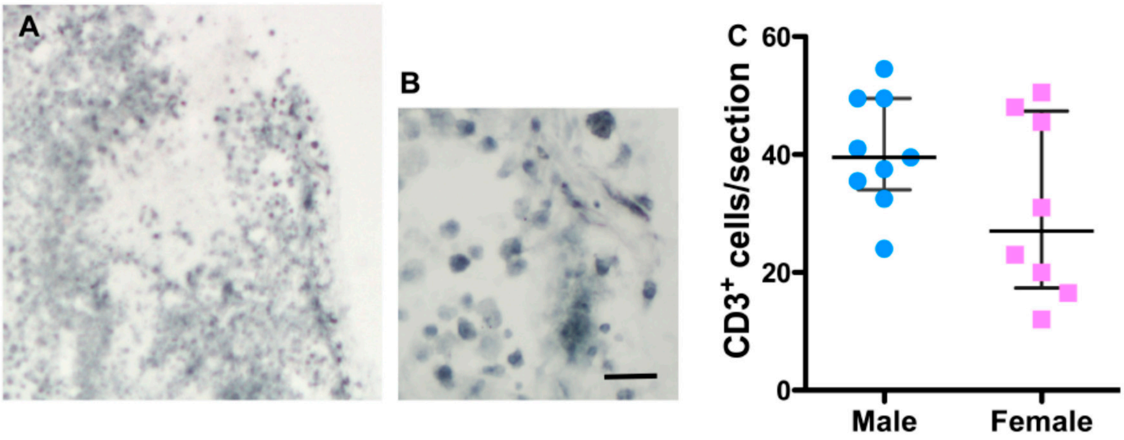

Figure S2. Presence of T lymphocytes 3 days after ischemia. (**A–B**): Representative images of $CD3^+$ lymphocytes staining in the lesion and leptomeningeal membranes. (**C**): Density of $CD3^+$ lymphocytes in male and female mice. Data are reported as median (25th – 75th percentiles).
